# Understanding Disclosure of Health Information to Workplace Friends

**DOI:** 10.3390/bs12100355

**Published:** 2022-09-23

**Authors:** Catherine Y. Kingsley Westerman, Emily M. Haverkamp, Cheng Zeng

**Affiliations:** Department of Communication, North Dakota State University, Fargo, ND 58102, USA

**Keywords:** communication privacy management, workplace friendship, social support

## Abstract

The purpose of this study was to learn about the process of disclosing health information to a coworker friend using the lens of Communication Privacy Management Theory. The study explores emerging themes regarding health information disclosure and predicts associations between privacy, social support, risk, stigma, and the willingness to disclose health information to a friend at work. Employees were asked to recall a time they shared health information with a coworker friend and report about the interaction via open-ended items and scales on a survey. The study found that as emotional support, instrumental support, perceived risk, and stigma of the information increased, so did the tendency to disclose to a coworker friend. Increased privacy of the information was associated with a decrease in the tendency to disclose. A thematic analysis of the open-ended results also revealed that employees shared information associated with personal on-going health problems to seek support, to relate to their coworker friends, and to maintain their friendship. The findings also indicated that employees were likely to receive social support from their coworker friends even if they were not seeking it.

## 1. Introduction

During the span of their working years, most people will experience a health-related situation that affects their ability to continue working, whether requiring a leave of absence, workplace accommodations, or support from others at their job. To give an idea of the scope of this issue: in January 2022, 7.8 million employees missed work due to an illness, injury, or medical problem or appointment, and 4.2 million worked only part time rather than full time due to illness, injury, or medical problem or appointment [1].

Employees have reasons to share information about their health at work, such as having people cover for them in their absence or obtaining accommodations that allow them to continue to work or return to work later. Westerman et al. [2] found that their participants would share health information at work for safety reasons (e.g., if their condition is contagious) or rumor prevention (e.g., to minimize ill will or gossip), or to gain social support at work, among others. However, employees who do decide to share their health information at work must contend with concerns about rejection, discrimination, loss of opportunity, loss of social support, and even the loss of employment (e.g., [2,3,4]). Because of these conflicting desires, employees are likely to experience dialectical tensions surrounding their decisions to disclose health information at work.

Coworker friends are an ideal potential confidant for employees because of their understanding of specific workplace dynamics as well as their ability to offer social support. However, employees may still experience the push–pull of dialectical tensions as they determine whether someone they consider a friend—but a coworker friend—is someone they can trust with their health information. Understanding these calculations better can help both employees who are making the decision to share or not to share and organizations who want to foster an open information environment to support employees and to maintain their ability to be successful. 

Communication Privacy Management Theory (CPM [5]) provides a dialectic framework to examine decisions about disclosing health information at work. CPM argues that individuals weigh risks and benefits of sharing private information with others as they make strategic decisions to share or withhold information. Previous research demonstrated that sharing health information is more likely when employees have high-quality relationships with supervisors [6] and good employee–organization relationships (EORs [7]). In this study, we are interested in another organizational relationship: coworker friends. 

Coworkers often become friends due to proximity, and friends at work can be an important source of both instrumental and emotional support [8]. Workers with increased autonomy and flexibility as well as increasingly adversarial relationships with organizations (e.g., [9,10]) may look to those they consider to be coworker friends for assistance with their health issues.

The goal of this study is to learn more about health disclosure interactions with coworker friends by triangulating qualitative and quantitative data. First, the study explores the decision to disclose health information to a coworker friend: what types of health issues do people share, why do people share, and what happens when people disclose to a coworker friend? Second, the study tests conceptual relationships drawn from CPM: how privacy of the information, the expectation of instrumental and emotional support, the perception of risk, and stigma of the health condition relate to the decision to disclose health information to coworker friends. The discussion then merges what we learned about CPM and health disclosure episodes from these two different approaches.

### 1.1. Workplace Friendship and Disclosure

Because of the close proximity people experience with their coworkers, the workplace is a natural site to begin, develop, and maintain friendships. Rumens [11] argues that although much research on workplace friendship focuses on organizational outcomes (e.g., improving productivity [12], reducing turnover [13]), workplace friendships have “personal and social significance…in their own right” [11] (p. 1151). That is, workplace friends may have value simply as friends rather than as a means to improving productivity. Workplace friendship is voluntary, has a personalistic focus, involves non-romantic ties, lacks exclusivity, and develops more slowly than other friendships [8]. A primary motivator of friendship is socio-emotional: friends should “foster affective and relational well-being” [14] (p. 3). Workplace friendships are considered special peer relationships as the companions experience high levels of intimacy, emotional support, and confirmation [8]. Friends at work can serve as a buffer to elements of work that people may not find desirable; they can also provide support or other intangible elements that organizations may not provide through official channels.

Workplace friendships also involve dialectic tensions, including a tension between openness and closedness [15]. Determining how much to share and with whom can be a difficult question, especially for health information disclosures. However, workplace friends may be attractive recipients of disclosure based on unique factors inherent to their role, such as their ability to provide social support combined with their heightened understanding of their coworker’s specific situation. Communication Privacy Management Theory (CPM [5]) helps to explain how people make decisions when managing dialectical tensions about sharing private information with others, such as workplace friends. CPM will be discussed next in relation to sharing health information with workplace friends.

### 1.2. Communication Privacy Management Theory

Communication Privacy Management Theory (CPM) explains the motivations, goals, and outcomes of sharing private information with others [5]. CPM focuses on how individuals manage private information in all contexts of daily life through the process of communication. Privacy management is a balancing act between revealing private information to receive some type of benefit such as support from others while maintaining the sense of autonomy that comes from keeping private information to oneself.

CPM [5] takes an episodic view because it focuses on discrete communicative instances as opposed to macro-level views of disclosure tendencies (e.g., Social Penetration Theory [16]) which examine disclosure in the context of relationship development. Research on disclosure episodes presumes that individuals engage in selective disclosing, influenced by a myriad of motivations as they regulate the information that they share and are reactive to recipient cues when deciding how, when, and with whom to reveal [5]. Sharing health information with a coworker friend is likely a common occurrence, but each episode may involve different calculations. CPM is particularly useful here because we are interested in employees’ selective disclosure given their consideration of various factors in specific episodes of disclosure to workplace friends. However, because this is a unique context for selective disclosure, the study first explores the episode for elements that fit into CPM structures as well as those that may not. With this goal in mind, the following research questions are posed:

RQ1: What types of health concerns are being shared with coworker friends?

RQ2: What influential factors are considered when disclosing health information to a coworker friend?

RQ3: How do coworker friends respond to a disclosure of personal health information?

CPM assumes that individuals perceive themselves as the owners of their private information. This ownership is often described as a metaphorical, or symbolic, boundary between public and private spheres wherein individuals believe they are entitled to certain levels of privacy. Privacy is the degree to which one is able to manage access to one’s information or to constrain how much personal information is shared with others [17,18]. Health information is particularly private [17,19], although some health information may be viewed as more private than other health information. For example, the degree of privacy may depend on whether the information shared is general (e.g., Bob is ill) or specific (e.g., Bob has a fungal infection), or on other elements such as the severity or contagious nature of an illness (e.g., [20,21]).

The degree to which private information must be guarded determines the quality of the boundary around that information. When the metaphorical boundary is thin or permeable, an individual is more likely to disclose their information. When a boundary is thick, or impermeable, the information is heavily guarded by that person, often in situations where the information is sensitive [5]. It is expected that willingness to disclose health information will vary as a function of privacy of the health information. That is, if the health information is seen as very private, the employee will be less likely to share it with their workplace friend. The following hypothesis is proposed:

**H1:**  *Perceived privacy of information will be negatively associated with willingness to disclose to a coworker friend.*

In the event individuals share their private information with others, those with whom they share become co-owners and hold a presumed shared responsibility to control the flow of said information. However, co-owners may knowingly or unknowingly mismanage another’s private information. To manage potential risks of private information sharing, individuals develop rules based on decision criteria to navigate co-ownership, to maintain privacy boundaries, and to regulate the transmission of their private information. According to CPM, decision-making criteria may include both fixed conditions (i.e., core criteria) and malleable conditions (catalyst criteria [22]). Some criteria hold more weight at times than others depending on personal needs or circumstances. 

When employees need to manage health information at work, they likely take into consideration both emotional needs and a risk–benefit analysis as disclosure decision criteria. Fulfilling emotional needs may lead employees to disclose information to a coworker they believe to be a potential source of support at work, particularly emotional support. When contemplating a risk–benefit analysis, employees might consider obtaining instrumental support as a benefit outcome of sharing. Employees may also consider privacy surrounding the information and stigma of the information as consequential risks of sharing. Lee and Li [7] found that perceived benefits of disclosing were strongly positively associated with the willingness to disclose information in organizations, for both physical and mental health. According to CPM, these decision criteria will affect the decision to disclose or withhold information.

### 1.3. Willingness to Disclose

Willingness to disclose is “how amenable people are to sharing their information” [6] (p. 1522). Managing disclosure decisions often requires weighing the consequences and benefits surrounding the disclosure, assessing the information to be disclosed, and evaluating the receiver of the information [23]. When employees are managing a health-related issue that affects their work, their willingness to disclose information to someone is important because they can potentially obtain the help they may need from someone in the workplace is by telling that person about the issue. If they can share with a coworker friend, they can benefit from the support provided by that coworker, whether it helps them manage stress, carry out their job better, or just feel supported at work. However, it is only based on self-disclosure that the recipient can understand that support is needed. Social support in different forms can be helpful to employees who are dealing with a health-related issue at work, and the expectation of receiving social support from a friend at work is likely to be part of the decision about disclosing. 

### 1.4. Social Support

During times of stress or uncertainty, people often rely on support from others to resolve or reappraise the challenges they perceive. Goldsmith [24] stated that what individuals do and say to assist each other broadly defines the concept of social support. Previous literature has conceptualized social support using both structural and functional components, recognizing its multidimensional nature along with its psychometric properties [21]. The structural elements of social support are often understood by the observable structures that surround support [25], such as the size of one’s network and how accessible that network is. The functional elements of social support are characterized by types of support, often organized by function and definition, such as in House’s [26] model. House [26] denotes four divisions of social support: emotional concern, instrumental aid, information assistance, and appraisal. These divisions have proven to be foundational in the development of modern social support models (see [27]) that extend and further define types of social support into five categories: information support, tangible support, esteem support, network support, and emotional support. However, two primary functional dimensions of social support (emotional and instrumental) consistently present as the most significant in previous literature [28]. Examining social support in terms of emotional and instrumental dimensions provides researchers with a general measure that can be used across contexts to better understand how support, obtainment of support, and ultimately disclosing the reason for needed support permeate various situations. 

Emotional support involves exchanges of empathy and messages conveying comfort, concern, and reassurance. Instrumental support is substantive help in completing a task or meeting a tangible need [24]. Emotional and instrumental support can both be given or received and they predominantly encompass all the types of social support (see [29]). When employees are dealing with challenges related to their health or the health of family members, they may seek emotional or instrumental support from workplace friends. Morelli, Lee, Arnn, and Zaki [30] found that between friends the provision of instrumental and emotional support benefitted both the receiver and the provider. However, the benefits of instrumental support were maximized when the provider was emotionally engaged while providing the support. This might indicate that the added elements of friendship, interdependence as co-workers, or an emotional component such as a health crisis may be crucial factors affecting social support and willingness to disclose to obtain that support. The connections between receiving and providing social support speak to the broader concepts of pro-social behavior and how people in close relationships help each other. 

Social support has been shown to lead to positive health outcomes such as enhanced stress recovery [31,32], improved psychological well-being [33,34], and physical health benefits that contribute to less instances of chronic illness and longer life expectancies [35,36]. Social support has also been linked to positive outcomes in the workplace. Previous research has found that employees seeking social support will be more likely to feel a sense of camaraderie amongst peers [37], have longer tenure at the organization [13], and experience greater levels of job satisfaction [38]. At the organizational level, social support moderates the effects of job satisfaction, increased productivity, organizational commitment, and various economic outcomes [39]. 

Obtaining social support often requires a disclosure in order to justify and articulate the type of support needed. A health disclosure decision, like most disclosure decisions, involves balancing the risks inherent to the disclosure with the potential benefits that may be gained from revealing. Coping with these dialectical dilemmas is expected to influence employees’ privacy rules such that they will disclose their health information to their coworker friends when they anticipate support will be forthcoming.

**H2:**  *The anticipation of emotional support will be positively associated with willingness to disclose to a coworker friend*.

**H3:**  *The anticipation of instrumental support will be positively associated with willingness to disclose to a coworker friend*.

### 1.5. Perception of Risk

The perception of risk is defined as “the degree to which one believes disclosing health information will lead to loss or harm” [6] (p. 1522). A sense of risk may emerge in the context of sharing health information at work because it may be inappropriate to disclose this type of information at work. More specifically, private disclosures may be interpreted as unprofessional in the workplace or perceived as placing undue burden on recipients of disclosures, particularly when information is deemed to be highly sensitive [40]. Westerman et al. [6] found that when the discloser’s relationship with their supervisor was of high-quality, the perception of risk was lower and the willingness to disclose was higher. Afifi and Steuber [41] state that relational quality and anticipated responses of the receiver are two factors that individuals consider when deciding whether to conceal or reveal private information and both have been positively related to willingness to disclose. The closer one feels towards their confidante, the more likely they are to expect a positive response to their disclosure, so disclosure is less risky, resulting in an increased likelihood to disclose to that person. 

Disclosures involve a pattern of selective sharing not limited to the information shared but also in the selection of the recipient of the disclosure. Most often, we choose the recipient of a disclosure based on a variety of factors including assessing the availability of recipients in each situation and evaluating them in a manner to minimize risk [23]. This study examines disclosure to coworker friends, who are generally people we trust. Given friends as recipients, the risk of sharing information should be low and willingness to disclose should be higher. However, if the risk is perceived to be higher, then willingness to disclose will be lower.

**H4:**  *Perceived risk will be negatively associated with willingness to disclose to a coworker friend*.

### 1.6. Stigma

Stigma is defined as a negative valuation placed on some quality a person or group of people has or is believed to have, such as a health condition. To be stigmatized is to be excluded because one does not behave in a socially acceptable way [42], or rather, that an individual is recognized for being different and devalued as such. According to Goffman [42] stigma does not reside in a person, but rather, stigma resides in the social context and occurs in social interactions. Stigma can manifest in many ways such as avoidance, aversion, social rejection, stereotyping, dehumanizing, and discrediting [43]. Stigma assigned to a characteristic or behavior may be further associated with other negative characteristics (e.g., smokers are seen as unproductive [44]), conflating the problem and promoting concealment. Stigmatized conditions are likely to be concealed at work (e.g., [45,46]), understandably given the possibility of exclusion and other negative organizational outcomes (e.g., [47,48]). Haslam et al. [45] found people to be unwilling or reluctant to share their mental health information at work, and Westerman et al. [6] found that when participants thought that their health conditions were highly stigmatizing, they were much less willing to share health information with their supervisor at work. Disclosure inherently involves vulnerability and potential risk; this risk is amplified when information is perceived to be stigmatized. However, despite the possible risks associated with stigmatized disclosures, people still choose to disclose stigmatized information. Based on CPM and previous work, people are likely to follow a highly restrictive protection rule for health information they believe to be stigmatizing.

**H5:**  *Stigma will be negatively associated with willingness to disclose to a coworker friend*.

### 1.7. Interactions with Catalyst Criteria

CPM predicts that individuals will weigh certain criteria differently in different situations. In this situation, it is expected that the two types of social support (emotional and instrumental) will be the main drivers of the willingness to disclose information for two reasons. First, one of the main functions of friendship is socio-emotional connection [14], so emotional support should be a strong predictor of disclosure. Second, another major reason to disclose health information at work is to receive help from another person [49,50], so instrumental support should also be a strong predictor. The overall support that coworker friends provide is distinct from that of other relationships because they possess shared knowledge and understanding, allowing them to appreciate workplace issues in a way that non-workplace friends cannot [51]. This makes them natural outlets when seeking organizational information or help. What is unclear is exactly how privacy of the information, risk, and stigma will enter into the disclosure calculations for coworker friends. We expect that social support will be the main driving force behind willingness to disclose to a coworker friend. Presuming that relationship is relatively strong, it is expected that the other criteria may strengthen or weaken the relationship between social support and willingness to disclose. The following RQs are posed:

RQ4: How will privacy, perception of risk, and stigma affect the relationship between the anticipation of emotional support and willingness to disclose to a coworker friend?

RQ5: How will privacy, perception of risk, and stigma affect the relationship between the anticipation of instrumental support and willingness to disclose to a coworker friend?

## 2. Materials and Methods

### 2.1. Participants

Upon receiving approval from the North Dakota State University IRB (#0004163), full-time employees were recruited to participate in the study via SurveyMonkey, which is an organization that assists with collecting specific samples. Participants were compensated USD 6 for their participation, and the average survey completion time was 8.7 min. There were 155 participants in the sample: 73 men and 82 women. Participants came from a variety of occupations and worked at organizations of different sizes (Micro-enterprise: *n* = 20, 12.9%; Small-enterprise: *n* = 26, 16.8%; Medium-enterprise: *n* = 38, 24.5%; Large-enterprise: *n* = 71, 45.8%). Participants’ organizational tenure varied from less than 1 year to 37 years (M = 8.44, SD = 8.13). About 40% of the respondents (*n* = 61) held a managerial position in their organizations. See Table 1 for more demographic information.

### 2.2. Procedures

Participants were asked to recall a time they shared health information with a coworker friend. A coworker friend was described to the participants as someone who was a coworker and also a friend, or a friend at work. We did not specify a hierarchical organizational level in the description; in other words, the coworker friend could be someone who was a peer or a supervisor or even an employee. Participants were given examples of health information ranging from a minor illness (e.g., flu, migraine) to an ongoing illness (e.g., cancer, depression), and invisible illnesses (e.g., anxiety) as well as family illnesses (e.g., parent illness) that might affect the focal individual’s ability to work. If they could not recall such a time, they were not allowed to continue the survey. In an effort to triangulate data, a mixed methods approach was implemented. Mixed methods was chosen to strengthen the overall picture of the phenomenon by providing us with an opportunity to both (1) test CPM ideas in the context of sharing health information at work and (2) explore elements of sharing health information at work that we may not have anticipated. The data will add to what is known about CPM as well as providing the opportunity to build new ideas about CPM in this context.

Those who did recall a time of sharing were first asked a series of open-ended questions pertaining to the disclosure event they recalled at work (e.g., What health information did you share? Why did you share your health information? How did your coworker/friend respond when you shared with them?). After answering the open-ended questions, participants responded to scales measuring CPM-related constructs pertaining to the situation they recalled.

### 2.3. Measures

#### 2.3.1. Privacy

Participants indicated their perception of the privacy of their health information via eight semantic differential items scaled from 1 to 7. This scale was previously used by Westerman et al. [6]. The sample items include “Exclusive-Unexclusive” and “Secret-Well-known”. Higher scores indicate that the information was perceived to be more private. The item “I consider the information I shared with my coworker/friend to be public (1)/private (7)” was dropped due to low factor loading. A confirmatory factor analysis (CFA) was performed on the scale. AMOS 28 was used to conduct all CFAs in this study. The goodness-of-fit statistics for the revised model were χ^2^ = 28.63, (*p* < 0.05); df = 14; goodness of fit index (GFI) = 0.95; comparative fit index (CFI) = 0.98; root mean square error of approximation (RMSEA) = 0.082, and SRMR = 0.047, demonstrating an acceptable fit. The internal consistency for this scale (Cronbach’s α) was 0.89.

#### 2.3.2. Perception of Risk

Participants indicated how risky they believed it was to share their information with their coworker friend via three Likert-type items modified from Bansal et al. [52] and scaled from 1 (very low) to 7 (very high). A sample item followed the lead-in “I believe that in giving health information to my coworker friend…. The risk involved was…(very low/very high)”. Higher scores indicate a greater perception of risk. A CFA was not conducted on this scale as a CFA on a measurement with only three items would yield a just-identified model with zero degrees of freedom. The internal consistency for this scale (Cronbach’s α) was 0.87.

#### 2.3.3. Social Support

Participants indicated the extent to which statements about receiving social support described their coworker friend. This social support scale [29] measured participants’ ratings of two types of social support: emotional support and instrumental support (treated as two subscales). Prior to deployment of the survey, this scale was modified in two ways: First, one item was dropped from the scale for emotional support as it did not directly pertain to an individual but referred to a “circle of people”. Second, the items were modified to identify the coworker friend as the focal individual. Six items measured emotional support (sample: I can talk to this person about the pressures in my life), and 4 items measured instrumental support (sample: This person would help me if I am physically unwell), on a Likert-type scale of 1(strongly disagree) to 7 (strongly agree). Higher scores indicate a perception of greater support from this individual. The goodness-of-fit statistics for the revised model were χ^2^ = 53.51, (*p* < 0.05); df = 34; goodness of fit index (GFI) = 0.94; comparative fit index (CFI) = 0.98; root mean square error of approximation (RMSEA) = 0.061, and SRMR = 0.041, demonstrating an acceptable fit.. The internal consistency for emotional support and instrumental support were, respectively, 0.92 and 0.76. 

#### 2.3.4. Social Stigma

The scale for social stigma (adapted from [53]) measured the extent to which the information shared was perceived as stigmatizing. Participants were requested to answer the questions about stigma pertaining specifically to the health information that they reported sharing. Participants responded to seven Likert-type items on a scale of 1 (strongly disagree) to 7 (strongly agree). A sample item is as follows: Most people feel that this health-related issue is something to be ashamed about. Higher scores indicate a greater degree of perceived stigma. The goodness-of-fit statistics for the revised model were χ^2^ = 30.61, (*p* < 0.01); df = 14; goodness of fit index (GFI) = 0.95; comparative fit index (CFI) = 0.98; root mean square error of approximation (RMSEA) = 0.088, and SRMR = 0.037, demonstrating an acceptable fit. The internal consistency for the scale (Cronbach’s α) was 0.91.

#### 2.3.5. Willingness to Disclose

The willingness to disclose scale was modified from [54] to include the coworker friend as the focal individual and included 5 Likert-type items scaled from 1 (strongly disagree) to 7 (strongly agree). A sample item is as follows: I felt compelled to tell someone at work about my health information. Higher scores indicate a greater willingness to disclose health information. After scrutinizing the factor loading of each item, the two items “I did not feel any need to tell anyone at work about my health information” and “I was willing to tell someone at work about my health information” with factor loadings lower than 0.5 were removed in an effort to purify the scale [55]. With only three items retained, a CFA was skipped as it would yield a just-identified model, which renders the result meaningless. The internal consistency for the revised scale (Cronbach’s α) was 0.79. 

### 2.4. Qualitative Analysis

The qualitative data were analyzed inductively, openly, and thematically using the iterative approach [56]. Iteration involved alternating between existing theories and emergent themes to draw conclusions. Participant answers were entered electronically for each of the open-ended questions about their health disclosure at work. Descriptive coding was completed independently by the researchers, who noted any emergent themes or connections in their research memos. First, the researchers described the participant responses in a general manner, such as by type of health issue, name of ailment, reason for disclosure, who the disclosure was made to, etc. Common responses were noted and eventually became preliminary themes within the data. Next, we conceptualized how responses could be organized categorically based on the initial typology described. Features of each answer helped organize the data into further common themes such as whether the ailment was noted as chronic or acute and whether the disclosure was based on an instrumental need or an emotional need. The researchers then met to compare their independent code lists and refine preliminary themes by agreeing on terminology and at what levels the data should be organized. Inferences were drawn as connections began to emerge and themes were finalized. The constant comparative method [57] was used to compare data to existing codes, consequently reworking codes to fit new data until the resulting list of themes was agreed upon. When codes were compared within the context of each answer and across the entire dataset the researchers were able to make claims that support and extend CPM theory. 

## 3. Results

### 3.1. Qualitative Results

Qualitative analysis revealed seven major themes that provide answers to RQs one, two, and three. The themes were grouped by research question and are described in further detail below. RQ1 asked about the type of health information shared between coworker friends; this is elucidated by the theme (1) sharing about health issues. RQ2 explored factors that were salient to participants when sharing about health information at work; these are represented by the themes (2) attributing disclosure to a quality of the confidant, (3) disclosing to save face, (4) disclosing to be social, and (5) disclosing to seek social support. Lastly, RQ3 was concerned with the outcomes after health information was shared with a coworker friend—the “aftermath;” these are represented by the themes (6) offering social support and (7) reciprocal sharing about health. 

### 3.2. RQ1: Types of Health Information 

The first research question asked what health information was being shared between friends at work. The type of health information participants described represented a variety of different concerns and health issues, ranging from minor illness to chronic health issues. 

#### Sharing about Health Issues

The health issues mentioned by participants were wide-ranging in keeping with our instructions, which were written to cast a wide net. Some types of health issues were more commonly mentioned. Participants shared about minor illnesses such as a cold, the flu, COVID-19 (experienced similarly to a cold), or just feeling ill. Other participants discussed ongoing health issues, which refer to disclosures about a health condition that is considered long-term, chronic, or has lasting effects. Some shared about health emergencies or cancer. To provide an overall picture of the types of health issues shared by the participants, the responses were categorized into eight groups with detailed subcategories (see Table 2). 

### 3.3. RQ2: Influential Factors of Disclosure

RQ2 determines the why of health information disclosures by examining what participants considered salient when deciding to reveal or conceal their health information. Specifically, RQ2 asked what influential factors were considered when sharing health information with co-worker friends. While many influencing factors seem to be at play, relational considerations emerged most prominently within the data when disclosing health information at work. The major themes associated with RQ2 are attributing disclosure to characteristics of the confidant, disclosing to save face, disclosing to be social, and disclosing to seek social support, all of which will be described below. 

#### 3.3.1. Attributing Disclosure to Characteristics of the Confidant 

Participants consistently attributed characteristics of their coworker friends as a reason why they disclosed. Characteristics of the confidant can be understood as any attribute of the coworker friend that prompted the participant to see them as an inviting recipient for disclosure. Trust and closeness were two recurring characteristics of the confidant that were described often in the data and invoked disclosures in this context. Participants described characteristics of their confidants in the following ways; “my friend is a good listener”, “I felt close to them”, “they are nice”, “I value their input”, “we share a bond”, and “my friend is trustworthy”. Assessing a confidant’s characteristics is in line with CPM theory which states individuals seek out qualities within their confidants that compel them to disclose private information to that person [5]. 

#### 3.3.2. Disclosing to Save Face

Disclosing to save face describes a health disclosure aimed at minimizing potential face threats to the participant, the confidant, and the relationship. Disclosing to minimize face threats to the participant looked like “I told them about my glass eye so they did not think I was being rude if they approached me on my blind side”, or “I needed to explain my time away [from work]”. Examples of disclosing to minimize face threats to the confidant were “they should know before everyone else” and “I owed it to my friend to be the one to tell them”. Lastly, disclosing to minimize face threats to the relationship were exemplified by answers such as “we cover for each other” and “I told them [in order to] to be upfront and honest”. 

#### 3.3.3. Disclosing to Be Social

Participants wrote about disclosing health information simply for the sake of “being social” with others at work. These participants were engaged in a social interaction with a friend; therefore disclosing was not necessarily a big decision. Disclosing to be social was observed in answers such as “I wanted to make conversation”, “because we were having lunch together”, “we were talking about our lives”, “it fit into conversation”, and quite explicitly to “be social”. 

#### 3.3.4. Disclosing to Seek Social Support

Disclosing to seek social support refers to participants describing that they shared about their health concern in the hopes of obtaining social support. Much of the support sought was emotional, but in some cases, it was instrumental. Instances of seeking emotional support are exemplified by these responses: “I was looking for advice”, “they went through this before”, “I trusted him to be supportive”, “I needed to talk”, and “I needed comfort”. Instances of needing instrumental support sounded like, “I knew they could help me stay on track”, “I needed to take time off”, and “they could help me prepare my team”. Our results suggested participants were looking mainly for emotional support but also instrumental support from their coworker friends. 

### 3.4. RQ3: Co-Worker Friend Response to Disclosure

The third research question inquired how coworker friends responded after participants disclosed their health information. The relevant themes demonstrated that recipients responded in two ways: with social support and by sharing their own relevant information.

#### 3.4.1. Offering Social Support

Overwhelmingly participants reported positive, supportive responses from their coworker friends. Confidants generally responded in an empathetic, sympathetic, and concerned manner. Their coworker friends provided emotional support, evident in frequent use of the words “empathy”, “sympathy”, “supportive”, “compassionate”, “understanding”, and “concerned” to describe coworker responses, as well as responses such as “they were concerned and asked me questions” and “they were concerned and offered me well-wishes”. In addition, it was common to receive offers of help from disclosure recipients; even if the participants did not explicitly seek instrumental help, coworker friends still offered it. Responses that captured offers of instrumental support included “they were empathetic and offered to help”, “they helped me by finding doctors”, and “they offered me a day off and checked to make sure I could work”. Overall, co-worker friends seemed to be supportive, concerned, and empathetic confidants. This theme revealed that not only did participants disclose in an effort to gain social support as described in answer to RQ2, they also received social support from their coworkers.

#### 3.4.2. Reciprocal Sharing about Health

Reciprocal sharing about health indicates that in response to a health information disclosure, participants experienced some type of reciprocal disclosure from their confidant. These responses demonstrate this reciprocity: “we discussed about their [the coworker’s] medical issue”, “[they] told me stories about family and friends that had the same problems”, “[she] shared her procedure with me”, “[they] said they could relate and were feeling it too”, and “[he] told me how it went for him”.

### 3.5. Quantitative Results

Pearson correlations were performed to test hypotheses 1–5 (see Table 3). The results revealed that perceived privacy was negatively related with willingness to disclose (r = −0.41, *p* < 0.01). The data were consistent with H1. Both emotional support (r = 0.29, *p* < 0.01) and instrumental support (r = 0.27, *p* < 0.01) were positively related with willingness to disclose. The data were consistent with H2 and H3. Perceived risk was found to be positively related with willingness to disclose (r = 0.19, *p* < 0.05). The data were not consistent with H4; rather, the discovered significant relationship was counter to the hypothesis. Stigma was positively related with willingness to disclose (r = 0.24, *p* < 0.01). The data were not consistent with H5 as the significant relationship was counter to the hypothesis. 

**Figure 1:**
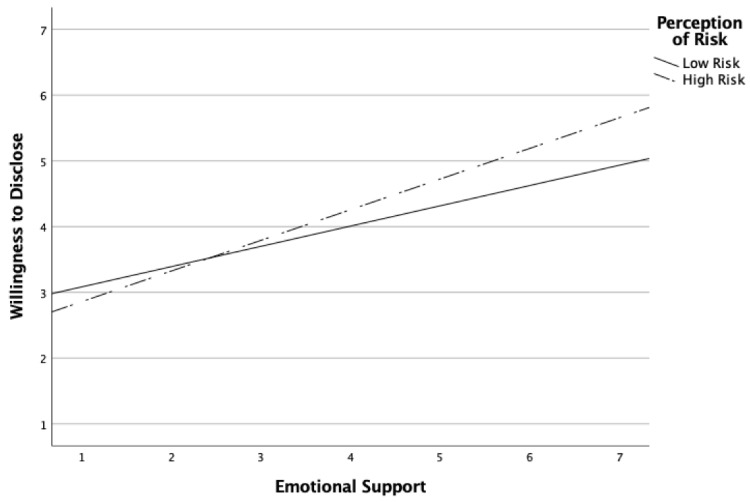
Perception of risk moderates the effect of emotional support on willingness to disclose.

**Figure 2:**
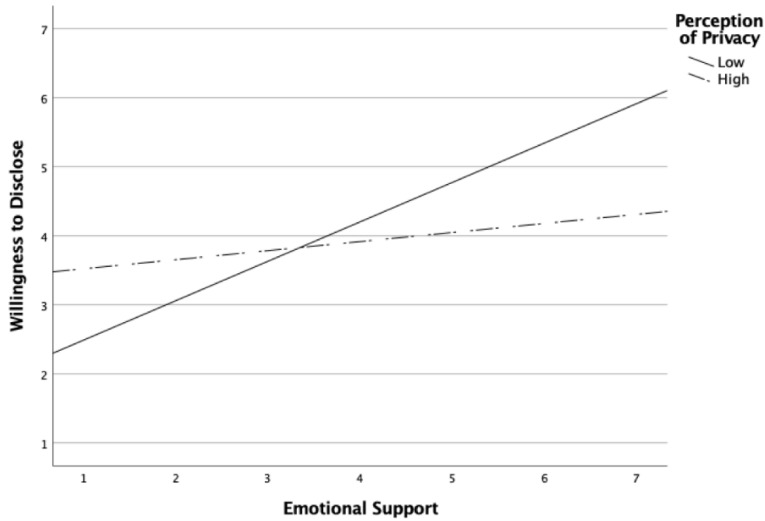
Perception of privacy moderates the effect of emotional support on willingness to disclose.

To address RQ4 and RQ5, Hayes’ [58] PROCESS was utilized to explore the moderating effects of privacy, perception of risk, and stigma on the relationships between emotional support and willingness to disclose, and between instrumental support and willingness to disclose. The moderation analyses yielded two significant results. First, perception of risk significantly moderated the relationship between emotional support and willingness to disclose. The bootstrap confidence interval for the moderation effect (b = 0.12, *p* < 0.05) was significant and entirely above zero (0.01 to 0.23), indicating the relationship between emotional support and willingness to disclose is stronger as the degree of risk increases. Second, privacy significantly moderated the relationship between emotional support and willingness to disclose. The bootstrap confidence interval for the moderation effect (b = −0.11, *p* < 0.05) was significant and entirely below zero (−0.217 to −0.0.02), indicating that the relationship between emotional support and willingness to disclose is weaker as the degree of privacy increases. Figure 1 and Figure 2 were added to help visualize the significant moderating effects.

## 4. Discussion

This study aimed to learn more about the decision to share health information at work with a workplace friend. Previous work has examined sharing with supervisors and organizations [6,7] but not with coworker friends in general. We collected both qualitative and quantitative data from each participant about a health disclosure experience. Emergent themes from the qualitative data combined with the findings of our quantitative analysis paint a picture that supports and extends the tenets of CPM theory, while also providing an additional explanatory layer to the claims made in this study. The results are discussed in more detail below.

### 4.1. Types of Health Information

The participants’ descriptions of their disclosure experience demonstrated a diversity of what is perceived to be a health issue. Previous work on disclosures often focused on specific health conditions such as HIV/AIDS status and diagnosis [53,59]; pregnancy-related disclosures including pregnancy loss [60] and infertility [61]; mental health disclosures about depression [62], eating disorders [63], and alcohol addiction [64]; as well as disclosures about breast cancer [65,66,67] and diseases such as Alzheimer’s [68]. Our data revealed a range of information participants shared with coworker friends. Although participants considered various elements in their decision to share, there was not much that was off-limits. People shared everything from a bipolar diagnosis to “My daughter’s attempts to commit suicide” with their coworker friends. 

We noticed a particular focus on sharing ongoing health conditions. Living with an ongoing health condition, or chronic illness, is a dynamic experience that often wavers between stigma concerns and normalization. Clair et al. [69] argue that normalization is a common coping strategy of those with chronic illness. It may be that sharing these ongoing health conditions with coworker friends is an attempt at normalizing chronic illness such that it seems commonplace and those with the ongoing condition are able to live, work, and be “normal”. Whereas some may consider long-term illnesses inherently private, those living with the ongoing health issue may desire a perception of normalcy; they may be disclosing to control the narrative in efforts to feel “normal”. Saving face may also be part of the effort to maintain normalcy in the face of chronic health conditions. Sharing with coworker friends ahead of time could help avoid awkward situations that could occur if the coworker friends were not aware of the health condition.

### 4.2. Predictions Based on CPM

Privacy of the information was negatively associated with the willingness to disclose to a coworker friend; if the information was highly private, then participants were less likely to share it. Based on CPM, it would be expected that information viewed as highly private would require more regulation. This finding is consistent with previous research [6] and CPM predictions that more private information requires maintaining more control over one’s information. The qualitative data indicated many of the health issues shared were chronic conditions or other health issues that would be difficult to conceal (e.g., pregnancy, cancer, pain, and absence from surgery). These conditions may not have been considered to be private because they could not be entirely hidden from others at work. Some disclosure would be inevitable, supporting the finding that less private health issues would be more likely to be disclosed. This may point to a need to consider the practicalities of disclosing when the health issue cannot be hidden and to look more closely at health issues that are visible versus invisible.

The participants’ assessment of the degree to which they expected their confidant to provide emotional support was positively associated with their willingness to disclose. This finding makes sense because one of the main functions of friendship is to provide affective and relational aid [14]. When participants expected that their coworker friend would be likely to provide that support, they were more likely to disclose. The qualitative data bolsters this finding, demonstrating that participants chose to disclose their health information because they were looking for emotional support. The qualitative data also extend our understanding beyond the initial CPM prediction. The expectation of emotional support linked to disclosing, and the qualitative data indicated emotional support was often given to those who disclosed. These findings are consistent with CPM predictions that emotional support will contribute to the decision to disclose. When people are looking for emotional support, it makes sense to seek it from a coworker friend by sharing about their health issues. It also appears coworker friends are willing to give emotional support.

Increasing privacy of health information weakened the relationship between emotional support and willingness to disclose. That is, if the information was seen as particularly private, the relationship between expectation of emotional support and willingness to disclose was weaker. This suggests that the privacy of the information is a highly influential factor. Even if a person expects they can get emotional support from their workplace friend, when their health information is very private, it dampens the likelihood that they will disclose the information to their friend.

The participants’ assessment of the degree to which they expected their confidant to provide instrumental support was also positively associated with the willingness to disclose. Given that one of the reasons to disclose to a workplace friend would be to get actual help doing a job, this makes sense. The qualitative data also indicated that participants were seeking instrumental support in some cases. Extending our understanding of how instrumental support fits into the picture, the qualitative data showed that participants received instrumental support from coworker friends, even when they did not specifically ask for it. These findings are consistent with the assertion that coworker friends can provide both emotional and instrumental support [8]. The findings are also consistent with CPM’s suggestion that people modify protection rules given different decision criteria—in this case, the need for instrumental support contributes to the willingness to disclose health information. Additionally, again, coworker friends seem more than willing to give instrumental support to those experiencing health-related issues.

Perceived risk was positively associated with willingness to disclose. This finding is inconsistent with Westerman et al. [6], who found that perceived risk demonstrated no relationship with willingness to disclose health information—but the study tested disclosure only to supervisors, which seems to be different from sharing with those considered to be coworker friends regardless of their hierarchical status. The finding is also inconsistent with CPM; we would expect a higher risk to be associated with greater reluctance to share. The qualitative data may be able to shed some light on this unusual finding. Although we did not find a specific theme about risk, some participants commented that they shared their health information because they believed it could be dangerous not to share. It could be that participants interpreted “risk” as how dangerous the condition could be if they did not reveal it, rather than how risky it was to share the information with a coworker friend. For example, one participant who disclosed they had diabetes, did it “so they would know in case something happens”. It could be that greater risk was a prompt to disclose for safety reasons.

The direct relationship between risk and disclosure was further qualified by an interaction between perceived risk and emotional support, such that when the risk was perceived to be higher, the relationship between emotional support and willingness to disclose became even stronger. The interaction helps make sense of the decision to disclose in the case of a situation where high risk was perceived and the participant expected to receive emotional support—with this combination, naturally the participant was more likely to share the information with their coworker friend. When the risk was believed to be greater, the need to share to receive emotional support was even stronger. This finding illustrates differential weighting of factors contributing to disclosure decisions, as discussed in CPM.

Stigma was positively associated with the willingness to disclose. This is inconsistent with the results found by Westerman et al. [6], who found a relatively strong negative relationship between stigma and willingness to disclose; however, their study focused on disclosure specifically to only supervisors rather than those considered to be coworker friends regardless of their hierarchical status. The finding is also inconsistent with CPM; we would expect a highly stigmatizing health condition to be associated with less permeable boundaries. However, the qualitative findings revealed that people were disclosing in an attempt to both save face and explain their conduct to others at work. This suggests that a more stigmatizing condition was actually a catalyst in the decision-making process, spurring people to disclose in an effort to minimize the stigma placed on them. Perhaps they were attempting to “get ahead” of any negative perceptions by sharing more stigmatizing conditions on their own terms. 

### 4.3. Responses to Disclosure

The qualitative findings revealed how coworker friends responded when health information was shared with them. These findings go beyond the predictions of CPM, extending our understanding of what happens after the decision to disclose to a coworker friend. What we learned was that the responses were similar to friendship outside of work in some ways but with an added component of understanding and ability to support. 

Coworker friends are in a unique position to offer support in part because of their friendship but also because of their familiarity with the surrounding circumstances. They know how others in the workplace will engage with the focal individual and may be able to relate in a unique way to the situation in which the focal individuals find themselves. In some ways, this may be similar to the “work spouse” relationship emerging in recent research [70], though likely less intense. Participants indicated they received emotional support readily and often from their confidants after they shared their health information. When you are friends, offering emotional support seems a natural response to disclosure of a health issue by a coworker.

Another theme that emerged regarding responses to disclosure was reciprocal sharing. Participants reported that when they shared their health situation, oftentimes their coworker friend would share back, either about having the same problem or going through the same procedure, or sharing other stories. Sharing equivalent disclosures is part of relational development according to Social Penetration Theory [16]. This type of response is offered in an effort to create connection and community between partners. Responding in this way is providing another form of emotional support to those confiding the information.

Finally, confidants also responded by offering instrumental support. Although participants for the most part were not necessarily seeking instrumental support, their confidants were quick to offer help, personal or professional. This may reflect a more problem-solving kind of orientation wherein people relate to each other by trying to help solve a problem.

### 4.4. Practical Implications

One practical implication is that a perceived closeness to others in the form of friendship at work can be beneficial, especially when you are managing a health issue. Our qualitative data revealed that when people disclosed to a coworker friend, they were “relieved”, “glad I shared”, and felt that “honesty is positive” and “people should be more open”. Practitioners should perhaps take a view similar to Rumens [11] and value workplace friendships not for their effects on productivity or turnover but for their intrinsic value. The value of coworker friendships simply as friendships is indicated by the importance of emotional support in our findings. Participants were both looking for and receiving emotional support associated with disclosing to their friend. This finding is in line with previous literature. Morelli, Lee, Arnn, and Zaki [30] assert that organizations should train individuals to enhance their emotional connections to one another, which will increase their ability to provide emotional support so they will reap maximum benefits. These findings suggest the benefits of receiving and giving social support over time by cultivating friendships in the workplace.

In addition to the intangible support, instrumental support from friends at work was also a contributor to helping to manage health information at work. Even when the participants were not specifically looking for instrumental support, often the response was to offer help. Overall, organizations may want to determine how they can facilitate and support coworker friendships. Not only does research demonstrate organizational benefits such as increased productivity, but it also demonstrates that workplace friendships are an important source of emotional and instrumental support when managing health information at work. Although we did not specify peer coworker friends, our findings strengthen and extend previous work on disclosure associated with the quality of supervisor relationships [6] and employee–organization relationships [7] by adding coworker friends to the mix of potential confidants.

One caveat for organizations to keep in mind: there can be some drawbacks to workplace friendships. Pillemer and Rothbard [14] propose some “dark sides” to workplace friendship. Particularly relevant here is their concern that receiving self-disclosure and providing socio-emotional support to friends at work can distract from the task at hand. Organizations may want to consider carefully how to manage the tension that coworker friends are likely to experience when there is a health-related issue for one or the other. They will need to balance the relief they may obtain from disclosing about health-related issues with the distraction the disclosure may present while at work. 

### 4.5. Limitations and Future Directions

One limitation may be the strong correlation between risk and stigma. Given what was learned in this study, future research should make an effort to improve the measurement to distinguish between these two factors or to identify other substantively different factors, such as the juxtaposition of hierarchical positions and friendship, which may be highly influential in the decision to share or withhold health-related information at work. 

Relatedly, another limitation of this research is our inability to distinguish the status dynamics of these coworker friendships. Because we did not specify peer friends, it is possible that participants reported on friends who were supervisors or who represented other types of hierarchical relationships. Future research should be careful to distinguish interest in peer coworker friendships versus friendships that involve supervisor–employee relationships. 

Looking at responses to disclosure of health information at work would be an interesting avenue for future research. Learning to what extent sharers are seeking particular outcomes from a disclosure (e.g., social support) and to what extent they actually achieve those outcomes in response could be informative as to how and why disclosure decisions are made. This would provide additional strength to our understanding of CPM by incorporating responses to disclosures as well as past conversational experiences. 

Some research on CPM [71] highlights the difference between individual privacy orientation and organizational privacy orientation. This may be influential in the decision to share health information at work, regardless of the partner. There were some indicators of this in our qualitative data. It was mentioned that people shared their health information because “everyone at work talks about this stuff”—this seemed to be specific to certain types of organizations, such as those in the healthcare industry. The type of organization and how they orient to privacy around health information as well as an individual’s own orientation to privacy are worth factoring in to future research in this area.

## Figures and Tables

**Table 1:** Participant demographics.

Variable	Range	*n*	M	SD
Sex				
Male		73		
Female		82		
Education				
Less than High School		13		
High school graduate		18		
Some College		40		
Bachelor’s degree		49		
Graduate degree		35		
Organizational Size				
Micro-enterprise (<10)		20		
Small enterprise (11–50)		26		
Medium enterprise (51–250)		38		
Large enterprise (>250)		71		
With a managerial position		61		
Weekly Working Hours	1–80		38.06	12.36
Tenure	0–37 yrs		8.44	8.13

**Table 2:** Categorized Summary of Health Information Shared in Open-Ended Questions.

Health Information Shared	*n*		Health Information Shared	*n*
**Cancer (subset of Chronic Condition)**	**7**		**Pregnancy-Related**	**9**
Cancer non-specific	3		Non-specific	4
Cancer Diagnosis	2		Pregnancy	2
Breast Cancer	1		Fertility issues	2
Thyroid Cancer	1		Miscarriage	1
**Health Emergency**	**13**		**COVID-Related**	**14**
Cancer-related emergency	4		Positive COVID test	5
Accidental injury emergency	3		COVID non-specific	5
Non-specific	3		COVID vaccine status	3
Appendectomy	1		Negative COVID test	1
Miscarriage Suicide Attempt	1 1		**Mental illness (subset of Chronic Condition)**	**15**
**Surgery**	**14**		Anxiety	4
Having surgical procedure (non-specific)	6		Depression Mental health (non-specific)	3 2
Vasectomy	2		Bipolar 2	1
Lung surgery	1		Difficulty Remembering	1
Lasik eye surgery	1		Suicide	1
Appendectomy	2		Eating Disorder	1
Mastectomy	1		Sexual Deviance	1
Tonsillectomy	1		Grief	1
**Chronic Condition**	**62**		**Other**	**21**
Mental Illness	15		Acute illness	11
Chronic disease non-specific Diabetes	12 7		Accidental Injury (non-emergency)	4
Cancer	7		Dental	2
Chronic pain	4		Body weight	2
Autoimmune non-specific	3		Medication symptoms	2
Sleep apnea	2			
Birth defect	1			
Irritable bowel disease	1			
Psoriatic Arthritis	1			
Asthma	1			
Heart problem	1			
GERD	1			
Diverticulitis	1			
Blind	1			
Lyme’s disease	1			
Hyperlipidemia	1			
Kidney disease	1			
Bunion	1			

**Table 3:** Alphas, Means, Standard Deviations, and Correlations of the variables ^1^.

Variable	*α*	(1)	(2)	(3)	(4)	(5)	(6)
(1) Willingness to Disclose	0.79	-					
(2) Perception of Risk	0.87	0.19 *	-				
(3) Perception of Privacy	0.89	−0.41 **	0.07	-			
(4) Emotional Support	0.92	0.29 **	−0.20 *	−0.06	-		
(5) Instrumental Support	0.76	0.27 **	−0.12	−0.14	0.69 **	-	
(6) Social Stigma	0.91	0.24 **	0.48 **	−0.06	−0.05	−0.04	-
M		4.47	2.53	4.26	5.04	4.77	2.95
SD		1.50	1.57	1.39	1.30	1.21	1.49

^1^ Note: * *p* < 0.05. ** *p* < 0.01.

## Data Availability

The data presented in this study are available from the corresponding author upon request. The data are not publicly available because participants were not informed that the data would be made publicly available.

## References

[1] Bureau of Labor Statistics 7.8 Million Workers Had an Illness-Related Work Absence in January 2022: The Economics Daily: U.S. Bureau of Labor Statistics. https://www.bls.gov/opub/ted/2022/7-8-million-workers-had-an-illness-related-work-absence-in-january-2022.htm.

[2] Westerman C.Y.K., Miller L.E., Reno K.M., Spates S.A. (2015). Sharing Personal Health Information at Work: What Is Appropriate and Expected in Organizations?. Commun. Stud..

[3] Munir F., Leka S., Griffiths A. (2005). Dealing with self-management of chronic illness at work: Predictors for self-disclosure. Soc. Sci. Med..

[4] Wheat K., Brohan E., Henderson C., Thornicroft G. (2010). Mental illness and the workplace: Conceal or reveal?. J. R. Soc. Med..

[5] Petronio S.S. (2002). Boundaries of Privacy: Dialectics of Disclosure.

[6] Westerman C.Y.K., Currie-Mueller J.L., Motto J.S., Curti L.C. (2017). How Supervisor Relationships and Protection Rules Affect Employees’ Attempts to Manage Health Information at Work. Health Commun..

[7] Lee Y., Queenie Li J.-Y. (2020). The value of internal communication in enhancing employees’ health information disclosure intentions in the workplace. Public Relat. Rev..

[8] Sias P.M., Cahill D.J. (1998). From coworkers to friends: The development of peer friendships in the workplace. West. J. Commun..

[9] Iacurci G. The Great Resignation Continues, as 44% of Workers Look for a New Job. https://www.cnbc.com/2022/03/22/great-resignation-continues-as-44percent-of-workers-seek-a-new-job.html.

[10] Kochan T.A., Yang D., Kimball W.T., Kelly E.L. (2019). Worker Voice in America: Is There a Gap between What Workers Expect and What They Experience?. ILR Rev..

[11] Rumens N. (2017). Researching workplace friendships: Drawing insights from the sociology of friendship. J. Soc. Pers. Relatsh..

[12] Song S.-H. (2006). Workplace Friendship and Employees’ Productivity: LMX Theory and the Case of the Seoul City Government. Int. Rev. Public Adm..

[13] Feeley T.H., Hwang J., Barnett G.A. (2008). Predicting Employee Turnover from Friendship Networks. J. Appl. Commun. Res..

[14] Pillemer J., Rothbard N.P. (2018). Friends Without Benefits: Understanding the Dark Sides of Workplace Friendship. Acad. Manag. Rev..

[15] Bridge K., Baxter L.A. (1992). Blended Relationships: Friends as Work Associates. West. J. Commun..

[16] Altman I., Taylor D.A. (1973). Social Penetration: The Development of Interpersonal Relationships.

[17] Alpert S.A. (2003). Protecting Medical Privacy: Challenges in the Age of Genetic Information: Protecting Medical Privacy. J. Soc. Issues.

[18] Stone E.F., Gueutal H.G., Gardner D.G., McClure S. (1983). A field experiment comparing information-privacy values, beliefs, and attitudes across several types of organizations. J. Appl. Psychol..

[19] Gostin L.O. (1994). Health Information Privacy. Cornell Law Rev..

[20] Liu Y., Canada K., Shi K., Corrigan P. (2012). HIV-related stigma acting as predictors of unemployment of people living with HIV/AIDS. AIDS Care.

[21] Vickers M.H. (1997). Life at work with “invisible” chronic illness (ICI): The “unseen”, unspoken, unrecognized dilemma of disclosure. J. Workplace Learn..

[22] Petronio S. (2013). Brief Status Report on Communication Privacy Management Theory. J. Fam. Commun..

[23] Greene K., Afifi T.D., Afifi W. (2015). An Integrated Model of Health Disclosure Decision-Making. Uncertainty, Information Management, and Disclosure Decisions.

[24] Goldsmith D.J. (2004). Communicating Social Support.

[25] Lindsey A.M., Yates B.C., Frank-Stromborg M., Olsen S.J. (2004). Social support: Conceptualization and measurement instruments. Instruments for Clinical Health-Care Research.

[26] House J.S. (1983). Work Stress and Social Support.

[27] Cutrona C.E., Suhr J.A. (1992). Controllability of Stressful Events and Satisfaction With Spouse Support Behaviors. Commun. Res..

[28] Declercq F., Vanheule S., Markey S., Willemsen J. (2007). Posttraumatic distress in security guards and the various effects of social support. J. Clin. Psychol..

[29] Shakespeare-Finch J., Obst P.L. (2011). The Development of the 2-Way Social Support Scale: A Measure of Giving and Receiving Emotional and Instrumental Support. J. Pers. Assess..

[30] Morelli S.A., Lee I.A., Arnn M.E., Zaki J. (2015). Emotional and instrumental support provision interact to predict well-being. Emotion.

[31] Goldberg S.G., Killeen M.B., O’Day B. (2005). The disclosure conundrum: How people with psychiatric disabilities navigate employment. Psychol. Public Policy Law.

[32] Priem J.S., Solomon D.H. (2015). Emotional Support and Physiological Stress Recovery: The Role of Support Matching, Adequacy, and Invisibility. Commun. Monogr..

[33] McGahey E., Waghorn G., Lloyd C., Morrissey S., Williams P.L. (2016). Formal plan for self-disclosure enhances supported employment outcomes among young people with severe mental illness: Managing personal information. Early Interv. Psychiatry.

[34] Holmstrom A.J., Russell J.C., Clare D.D. (2013). Esteem Support Messages Received during the Job Search: A Test of the CETESM. Commun. Monogr..

[35] Levula A., Wilson A., Harré M. (2016). The association between social network factors and mental health at different life stages. Qual. Life Res..

[36] Thoits P.A. (2011). Mechanisms Linking Social Ties and Support to Physical and Mental Health. J. Health Soc. Behav..

[37] Cranmer G.A., Goldman Z.W., Booth-Butterfield M. (2017). The Mediated Relationship Between Received Support and Job Satisfaction: An Initial Application of Socialization Resources Theory. West. J. Commun..

[38] Raile A.N.W., Kim R.K., Choi J., Serota K.B., Park H.S., Lee D.W. (2008). Connections at Work: How Friendship Networks Relate to Job Satisfaction. Commun. Res. Rep..

[39] Hochwarter W.A., Kacmar C., Perrewé P.L., Johnson D. (2003). Perceived organizational support as a mediator of the relationship between politics perceptions and work outcomes. J. Vocat. Behav..

[40] Charoensap-Kelly P., Mestayer C.L., Knight G.B. (2020). To Come Out or Not to Come Out: Minority Religious Identity Self-Disclosure in the United States Workplace. Manag. Commun. Q..

[41] Afifi T.D., Steuber K. (2010). The cycle of concealment model. J. Soc. Pers. Relatsh..

[42] Goffman E. (1963). Stigma: Notes on the Management of Spoiled Identity, 1st Touchstone ed..

[43] Herek G.M. (1999). AIDS and Stigma. Am. Behav. Sci..

[44] Roulin N., Bhatnagar N. (2018). Smoking as a Job Killer: Reactions to Smokers in Personnel Selection. J. Bus. Ethics.

[45] Haslam C., Atkinson S., Brown S., Haslam R. (2005). Anxiety and depression in the workplace: Effects on the individual and organisation (a focus group investigation). J. Affect. Disord..

[46] Gray R.E., Fitch M., Phillips C., Labrecque M., Fergus K. (2000). To tell or not to tell: Patterns of disclosure among men with prostate cancer. Psychooncology.

[47] Phelan J.C., Link B.G. (2004). Fear of People with Mental Illnesses: The Role of Personal and Impersonal Contact and Exposure to Threat or Harm. J. Health Soc. Behav..

[48] Puhl R., Brownell K.D. (2001). Bias, Discrimination, and Obesity. Obes. Res..

[49] Saks A.M., Gruman J.A., Connie Wanberg R. (2012). Getting Newcomers On Board: A Review of Socialization Practices and Introduction to Socialization Resources Theory. The Oxford Handbook of Organizational Socialization.

[50] Hobfoll S.E. (2002). Social and Psychological Resources and Adaptation. Rev. Gen. Psychol..

[51] Sias P. (2009). Organizing Relationships: Traditional and Emerging Perspectives on Workplace Relationships.

[52] Bansal G., Zahedi F., Gefen D. (2010). The impact of personal dispositions on information sensitivity, privacy concern and trust in disclosing health information online. Decis. Support Syst..

[53] Derlega V.J., Winstead B.A., Greene K., Serovich J., Elwood W.N. (2004). Reasons for HIV Disclosure/Nondisclosure in Close Relationships: Testing a Model of HIV–Disclosure Decision Making. J. Soc. Clin. Psychol..

[54] Motto J.S. (2013). Speaking of Gay: An Exploratory Study of the Influence of Privacy Management on Parents’ Disclosures of a Son’s Or Daughter’s Sexual Orientation. Ph.D. Thesis.

[55] Hinkin T.R. (1998). A Brief Tutorial on the Development of Measures for Use in Survey Questionnaires. Organ. Res. Methods.

[56] Tracy S.J. (2020). Qualitative Research Methods: Collecting Evidence, Crafting Analysis, Communicating Impact.

[57] Charmaz K. (2014). Constructing Grounded Theory.

[58] Hayes A.F. (2018). Partial, conditional, and moderated moderated mediation: Quantification, inference, and interpretation. Commun. Monogr..

[59] Haas S.M., Perazzo J.D., Ruffner A.H., Lyons M.S. (2020). Exploring Current Stereotypes and Norms Impacting Sexual Partner HIV-Status Communication. Health Commun..

[60] Steimel S. (2021). Communication Privacy Management and Pregnancy Loss in Interpersonal Workplace Communication. Womens Stud. Commun..

[61] Bute J.J. (2013). The Discursive Dynamics of Disclosure and Avoidance: Evidence from a Study of Infertility. West. J. Commun..

[62] Meluch A.L., Starcher S.C. (2020). College Student Concealment and Disclosure of Mental Health Issues in the Classroom: Students’ Perceptions of Risk and Use of Contextual Criteria. Commun. Stud..

[63] Herrman A.R., Tenzek K.E. (2017). Communication Privacy Management: A Thematic Analysis of Revealing and Concealing Eating Disorders in an Online Community. Qual. Res. Rep. Commun..

[64] Romo L.K., Dinsmore D.R., Watterson T.C. (2016). “Coming out” as an alcoholic: How former problem drinkers negotiate disclosure of their nondrinking identity. Health Commun..

[65] Weber K.M., Solomon D.H. (2008). Locating Relationship and Communication Issues Among Stressors Associated With Breast Cancer. Health Commun..

[66] Lillie H.M., Venetis M.K., Chernichky-Karcher S.M. (2018). “He would never let me just give up”: Communicatively Constructing Dyadic Resilience in the Experience of Breast Cancer. Health Commun..

[67] Wellman M.L., Holton A.E., Kaphingst K.A. (2022). Previvorship Posting: Why Breast Cancer Previvors Share Their Stories on Social Media. Health Commun..

[68] Miller-Ott A.E. (2020). “Just a Heads Up, My Father Has Alzheimer’s”: Changes in Communication and Identity of Adult Children of Parents with Alzheimer’s Disease. Health Commun..

[69] Clair J.A., Beatty J.E., Maclean T.L. (2005). Out of Sight But Not Out of Mind: Managing Invisible Social Identities in the Workplace. Acad. Manag. Rev..

[70] McBride M.C., Bergen K.M. (2015). Work Spouses: Defining and Understanding a “New” Relationship. Commun. Stud..

[71] Frampton B.D., Child J.T. (2013). Friend or not to friend: Coworker Facebook friend requests as an application of communication privacy management theory. Comput. Hum. Behav..

